# Extracts from *Curcuma zedoaria* Inhibit Proliferation of Human Breast Cancer Cell MDA-MB-231 *In Vitro*

**DOI:** 10.1155/2014/730678

**Published:** 2014-05-05

**Authors:** Xiu-fei Gao, Qing-lin Li, Hai-long Li, Hong-yan Zhang, Jian-ying Su, Bei Wang, Pei Liu, Ai-qin Zhang

**Affiliations:** ^1^The First Affiliated Hospital of Zhejiang Chinese Medical University, Hangzhou, Zhejiang 310006, China; ^2^Zhejiang Cancer Hospital, Zhejiang 310022, China

## Abstract

*Objective.* To evaluate the effect of petroleum ether extracts of *Curcuma zedoaria* on the proliferation of human triple negative breast cancer cell line MDA-MB-231. *Methods.* The reagents were isolated from *Curcuma zedoaria* by petroleum ether fraction. It was assayed by CCK8 for MDA-MB-231 cellular viability with various concentrations and days, cell cycle analyses, Western Blot analysis, and Realtime Reverse Transcriptase PCR analyses for chemokines molecules including E-cadherin, and E-selectin, and adhesion molecules including CCR7, SLC, SDF-1, and CXCR4. Epirubicin was used as control in the study. *Results.* MDA-MB-231 cells were inhibited by petroleum ether extracts of *Curcuma zedoaria* (*P* < 0.05), and the inhibition rate was dependent on concentrations and time. Petroleum ether extracts of *Curcuma zedoaria* as well as Epirubicin produce a significant G0/G1 cell cycle arrest. The level of expression of proteins E-cadherin and E-cadherin mRNA was significantly increased, while proteins SDF-1, CCR7, and CXCR4 mRNA were decreased after being incubated with petroleum ether extracts of *Curcuma zedoaria* at the concentrations of 300 **μ**g/mL than control (*P* < 0.05). The differences were that the protein CXCR4 mRNA expression level was higher than vehicle. *Conclusions.* MDA-MB-231 cells were inhibited by petroleum ether extracts of *Curcuma zedoaria*.

## 1. Introduction

Breast cancer (BC) is one of the most common human malignancies, accounting for 22% of all cancers diagnosed in women. BC represents a complex and heterogeneous disease comprising distinct pathologies with specific histological features, therapeutic responses, metastatic dissemination patterns, and patient outcomes. In recent years, global molecular analyses have revealed four main distinct subgroups in human breast tumors: luminal A and luminal B (LA and LB), human epidermal growth factor receptor 2-overexpressing (Her2), and triple-negative breast cancer (TNBC) [1]. Of all the BC subgroups, TNBC is accounting for 10%~20.8% [2]. TNBC represent the greatest clinical challenge because these tumors are prevalent in younger women, associated with the worst prognosis and often relapse rapidly [3]. In contrast to nontriple-negative breast cancer, TNBC are often highly histological grade and malignancy, and preferentially metastasize to lung, liver, and brain [4]. TNBC is one of the worst prognosis in BC subgroups [5]. ER-positive luminal tumors and Her2 carcinomas, which can be treated with targeted therapies such as Tamoxifen (estrogen antagonist), aromatase inhibitors, or anti-Her2 monoclonal antibodies [6]; there is no available targeted therapy for TNBC. Patients with TNBC are treated exclusively with conventional chemotherapy. While they show high rates of objective initial response, the majority of patients do not have a complete and prolonged response and are at high risk for relapse and death within the first 3~5 years of diagnosis [7]. Epirubicin is extensively used in chemotherapy for patients with breast cancer. In spite of its excellent antitumor activity, the associated acute and chronic toxicities lead to a relatively low therapeutic efficacy. Whereas, some molecules are in clinical trials in patients with TNBC, such as dasatinib (Src inhibitor), cetuximab (EGFR inhibitor), bevacizumab (vascular endothelial growth factor inhibitor), or olaparib (Poly [ADP-ribose] polymerase inhibitor) [8]; identification of relevant molecular targets in TNBC remains a critical challenge.

Accumulated data have demonstrated that complementary and alternative medicine (CAM) show beneficial effect in the treatment of several kinds of cancers [9–11]. Curcuma is a commonly prescribed Chinese herb with anticancer potentials. Curcuma extracts and active ingredients have been identified as its main bioactive components with anticancer effect both* in vitro* and* in vivo* [12, 13]. Furanodiene significantly inhibited cancer cell proliferation while germacrone and curdione showed no effect. Germacrone enhanced furanodiene's antiproliferative effect; curdione showed no effect on furanodiene's antiproliferative effect but partly reversed the antiproliferative effect of germacrone and furanodiene combined. There are unpredictable and complex interactions among different components in curcuma phaeocaulis. Therefore, in this study, petroleum ether extracts of this plant, not isolated sesquiterpene, has effect on the proliferation of human triple negative breast cancer cell line MDA-MB-231* in vitro*.

## 2. Materials and Methods

### 2.1. Chemicals

RPMI 1640, fetal bovine serum (FBS), phosphate-buffered saline (PBS), TRIzol, SuperScript reverse transcriptase enzyme, and buffer were purchased from Gibco-BRL Company (USA). Dimethyl sulfoxide (DMSO), DEPC, and propidium iodide (PI) were purchased from Sigma-Aldrich Chemical Company (USA). CCK-8 Kit was purchased from DOJINDO Company (Japan). Ethanol, petroleum ether, ethyl acetate, n-butanol, and all other chemicals were of analytical grade. Target gene primers and probes were synthesized from Takar Company. Taq DNA polymerase and buffer were purchased from Roche Company. The antibodies for detecting E-cadherin, E-selectin, CCR7, SLC, SDF-1, and CXCR4 were purchased from Cell Signaling Technology.

### 2.2. Cell Culture

MBA-MB-231 cells were cultured in RPMI 1640 medium supplemented with 10% FBS and 40 mg/L gentamicine. Cells were maintained at 37°C in a humidified atmosphere containing 5% CO_2_.

### 2.3. Plant Material and Extraction Procedure

*Curcuma zedoaria* were collected from Guangxi province (China) in September, 2012. They were deposited in the First Affiliated Hospital of Zhejiang Chinese Medical University. The identity of the plant was confirmed by morphological examination in comparison to the herbarium specimens.

The sample was pulverized to 40 meshes. The sample (40 g) was weighed accurately and placed into a 100 mL flask containing 400 mL 70% ethanol, then soaked overnight. The sample was extracted two times through heating reflux. The ethanol extracts were filtered and concentrated under reduced pressure to give a residue. Then the residue was extracted with petroleum ether. The petroleum ether extracts were dried with rotary evaporators to obtain the dried petroleum ether extracts. The 5 mg of petroleum ether extracts was dissolved in 500 *μ*L DMSO to prepare a stock solution of 10 mg/mL. The stock solution was stored at −20°C until use.

### 2.4. *In Vitro* CCK8 Assay for Cellular Viability

Cell counting kit-8 (CCK-8 reagent) can be used for simple and accurate cell viability assay. The basic principle is the reagent containing WST-8 (chemical name: 2-(2-methoxy-4-nitrophenyl)-3-(4-nitrophenyl)-5-(2,4-disulfonic, acid phenyl)-2H- tetrazolium, monosodium salt) is reduced to Formazan dye by a dehydrogenase enzyme of cell mitochondria through electron carrier 1-Methoxy PMS.MDA-MB-231 cells which were seeded at a density of 1 × 10^4^ cells per 200 *μ*L per well in 96-well microtiter plates (Promega Corporation). 24 h after seeding, the cultures were washed twice with PBS and then exposed to various concentrations of petroleum ether extracts of* Curcuma zedoaria* (100 *μ*g/mL, 200 *μ*g/mL, 300 *μ*g/mL, 400 *μ*g/mL, 500 *μ*g/mL), various concentrations of Epirubicin (0.25 *μ*g/mL, 0.5 *μ*g/mL, 1 *μ*g/mL, 2 *μ*g/mL, 4 *μ*g/mL), and DSMO as vehicle for 24 h or 5 days. Per concentration was performed in triplicate. Then, 10 *μ*L of CCK8 solution was added to each well, and the plates were incubated for an additional 3 hours at 37°C. Cell viability was measured as the absorbance at 450 nm with a microplate reader (Synergy 2 Multimode Microplate Reader). BioTek, Winooski, VT (USA) and expressed as a percentage of the control level. The mean optical density (OD) values from triplicate wells for each treatment were used as the index of cell viability.

### 2.5. Cell Cycle Analyses

MDA-MB-231 cells were plated in 24-well plates and maintained in 10% FBS RPMI1640 at a density of 1 × 10^6^ cells for 24 hours. Cells were then exposed to petroleum ether extracts of* Curcuma zedoaria* (300 *μ*g/mL), Epirubicin (1 *μ*g/mL), DSMO as control group, grown another 24 hours. Cells were harvested by trypsinization and pelleted by centrifugation. The pellets were then resuspended in PBS containing 50 *μ*g/mL propidium iodide, 0.1% Triton X-100, and 0.1% sodium citrate. Propidium iodide fluorescence was measured by fluorescence-activated cell sorting by flow cytometry (FACS Canto II, Becton Dickinson, USA) using the Multicycle's cycle analysis software.

### 2.6. Western Blot Analysis

MDA-MB-231 cells were washed once in PBS supplemented with complete protease inhibitor (Roche, Mannheim, Germany). Washed cell pellets (3 × 10^5^ cells) were resuspended in protease buffer containing 10 mM Tris-buffer (pH 7.6), 1.5 mM MgCl_2_, 1 mM EDTA, 10 mM KCl, 1 mM phenylmethylsulphonyl fluoride (PMSF), and protease inhibitor tablets (contain 4-(2-aminoethyl) benzenesulfonyl fluoride (AEBSF), E-64, bestatin, leupeptin, aprotinin, and EDTA for inhibition of serine, cysteine, and metalloproteases). Whole cell extracts were prepared according to the published methods [14]. Briefly, MDA-MB-231 cells were washed once in cold PBS, followed by the cell pellets which were lysed by a single freeze-thaw cycle in the presence of protease inhibitors and whole cell extracts were obtained by centrifugation at 14000 rpm for 40 min after extraction with 0.5 M NaCl. Protein concentrations were determined using the Bio-Rad microprotein assay using bovine serum albumin as the standard. Twenty-five microgram of each protein sample was resolved by 10 or 12% SDS-PAGE and electroblotted onto nitrocellulose membranes (Bio-Rad, Hercules, CA, USA). The membranes were blocked for 1 h at room temperature in PBS containing 5% skim milk plus 0.1% Tween-20 (PBST) and incubated overnight at 4°C with different first antibodies, followed by incubation with a horseradish-peroxidase-conjugated secondary antibody (Jackson ImmunoResearch, West Grove, PA, 1 : 10000 dilution) for 1 hour at room temperature.

### 2.7. Realtime Reverse Transcriptase PCR Analyses

Cellular total RNA was extracted with TRIzol reagent (Invitrogen). The RNA concentration and purity were measured using a spectrophotometer. cDNA was synthesized from total RNA by reverse transcription. The primer sequences and product size were as follows. E-cadherin forward: 5′-CGG GAA TGC AGT TGA GGA TC-3′, reverse: 5′-AGG ATG GTG TAA GCG ATG GC-3′, E-selectin forward: 5′-TGC ATG GAG GGT TGT TAA TGG-3′, reverse: 5′-GGA TGA A AG TGA TTA AAT TGT GCA TAG-3′, CCR7 forward: 5′-TGC CAT CTA CAA GAT GA GCT-3′, reverse: 5′-GGT GCT ACT GGT GAT GTT GA-3′, CXCR4 forward: 5′-GCT GTT GGC TGA AAA GGT GGT C-3′, reverse: 5′-CAC CTC GCT TTC CTT TGG AGA-3′, SDF-1 forward: 5′-CAG CCG TGC AAC AAT CTG AAG-3′, reverse: 5′-CTG CAT CAG TGA CGG TAA ACC-3′, SLC forward: 5′-TCC CGG CAA TCC TGT TCT C-3′, reverse: 5′-CCT TCC TCA GGG TTT GCA CA-3′, GAPDH forward: 5′-CAG CCT CAA GAT CAT CAG CA-3′, reverse: 5′-ACA GTC TTC TGG GTG GCA GT-3′. GAPDH was used as an internal control. The products were checked by agarose electrophoresis and analyzed using Fast Realtime PCR system (ABI, USA).

### 2.8. Statistical Analysis

Petroleum ether extracts of* Curcuma zedoaria* were observed object, DMSO as vehicle and Epirubicin as positive control during all experiments. Each viability value represents the mean ± SD. From three determinations, IC50 values were calculated from the log-log plot between the percentages of viable cells. Subsequently, each experiment was performed in triplicate measurements. Statistical analysis of data was carried out using a one-way ANOVA followed by Holm-Sidak pairwise multiple comparison test (Sigma Plot, Systat Software Inc.), and a probability value of less than 0.05 (*P < 0.05, **P < 0.01) was accepted as a significant difference.

## 3. Results

### 3.1. *In Vitro* CCK8 Assay for Cellular Viability

The results from the* in vitro* CCK8 assay of cellular viability in various concentrations of petroleum ether extracts of* Curcuma zedoaria* as well as the Epirubicin are listed in Table 1. MDA-MB-231 cells were inhibited by petroleum ether extracts of* Curcuma zedoaria* (*P* < 0.0), and the inhibition rate was higher with increasing concentration (Figure 2(a)). The inhibition rate of petroleum ether extracts of* Curcuma zedoaria* (500 *μ*g/mL) is about 90%, and there are little viable cells in electron microscope. These relationships were measured graphically by plotting concentrations versus percentage of inhibition. According to Table 1, the inhibition rate of petroleum ether extracts of* Curcuma zedoaria* (300 *μ*g/mL) is more than 50%, then the following experiments were performed with petroleum ether extracts of* Curcuma zedoaria* (300 *μ*g/mL) and Epirubicin (1.0 *μ*g/mL) of middle concentration. The cell grow curve shows that the inhibition of cell proliferation is higher with time grew by petroleum ether extracts of* Curcuma zedoaria* on 300 *μ*g/mL concentration (Figure 2(b)). Under the microscope, on first day, the cells had microscopic contour increase and continued to proliferate. On the second and third days, cell proliferation was slower, cell outline was clear, and cytoplasm was rough; there were particle accumulations. On the fourth day, the cytoplasm was extremely rough with much debris inside. On the fifth day, cell accumulated and many cells were necrotic (Figure 2(c)).

### 3.2. Cell Cycle Analyses

The analysis of cell cycle phase distribution demonstrated that petroleum ether extracts of* Curcuma zedoaria* as well as Epirubicin produce a significant increase in the number of MDA-MB-231 cells in G1 phase at 24 h after treatment, clearly demonstrating a significant G0/G1 cell cycle arrest (Table 2). A consequent decrease in cells in S and G2 phase versus control was also observed. (Figures 3(a) and 3(b)).

### 3.3. Western Blot Analysis

It is known that chemokines and adhesion molecules family are strongly linked to the process of tumor recurrence and metastasis. Some members of the family are chemokines molecules such as E-cadherin and E-selectin, and adhesion molecules such as CCR7, SLC, SDF-1, and CXCR4. We determined the expression of these proteins by Western blot. We showed that 24 h after treatment with petroleum ether extracts of* Curcuma zedoaria*, the level of expression of protein E-cadherin and CXCR4 were significantly increased, while protein SDF-1 was decreased in relation to control at the same time. The level of expression of protein E-selectin was slightly increased and protein CCR7 was slightly decreased, although this increase was not statistically significant. The difference is that the level of expression of proteins E-cadherin, E-selectin, and SDF-1 was significantly decreased, while protein CXCR4 was increased in relation to control at the same time after treatment with Epirubicin. (Figures 4(a) and 4(b)).

### 3.4. Realtime Reverse Transcriptase PCR Analyses

Reverse transcriptase-polymerase chain reaction (RT-PCR) analysis demonstrated that the E-cadherin mRNA expression level was higher and the CCR7 and CXCR4 mRNA expression level were lower 24 h after incubated with petroleum ether extracts of* Curcuma zedoaria* at the concentrations of 300 *μ*g/mL than control (*P* < 0.05). The level of expression of protein E-cadherin was significantly increased, while protein CXCR7 was decreased in relation to control at the same time after treatment with Epirubicin. On the other hand, the difference is that the CXCR4 mRNA expression level was higher and the SDF-1 mRNA expression level was lower. (Table 3, Figures 5(a) and 5(b)).

## 4. Discussion

**Figure 1:**
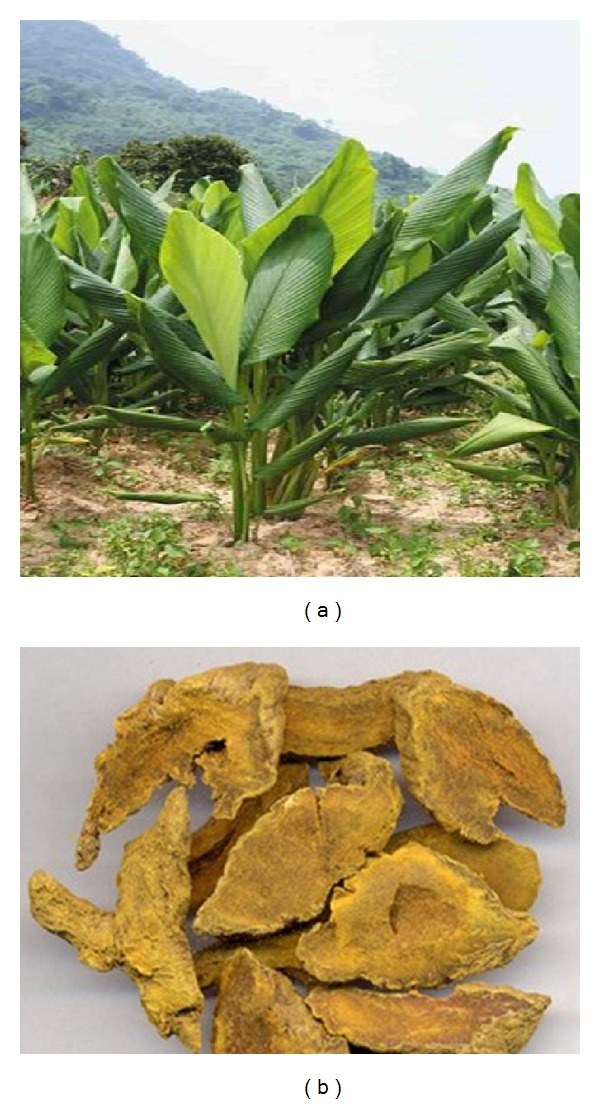
*Curcuma zedoaria* of the plant and the plant root sample.

**Figure 2:**
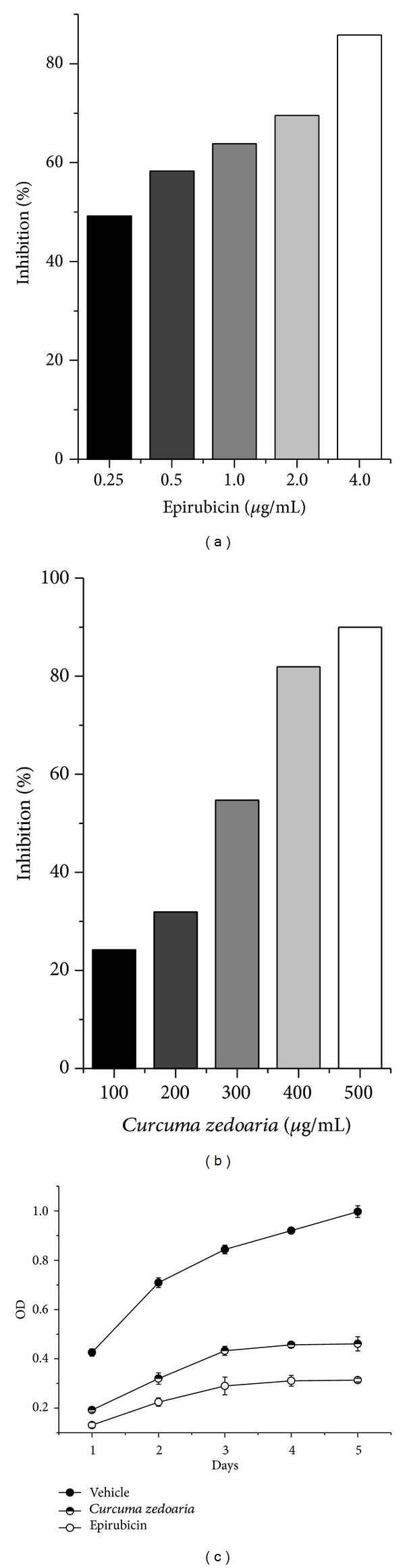
The inhibition rate of the various concentrations of epirubicin (a) and petroleum ether extracts of* Curcuma zedoaria* (b) were determined by CCK8 assays. The values were reported as %. (c) The inhibition rate of different days was determined by CCK8 assay as described compared with vehicle. Values are average of triplicate experiment and are represented as mean ± SD.

**Figure 3:**
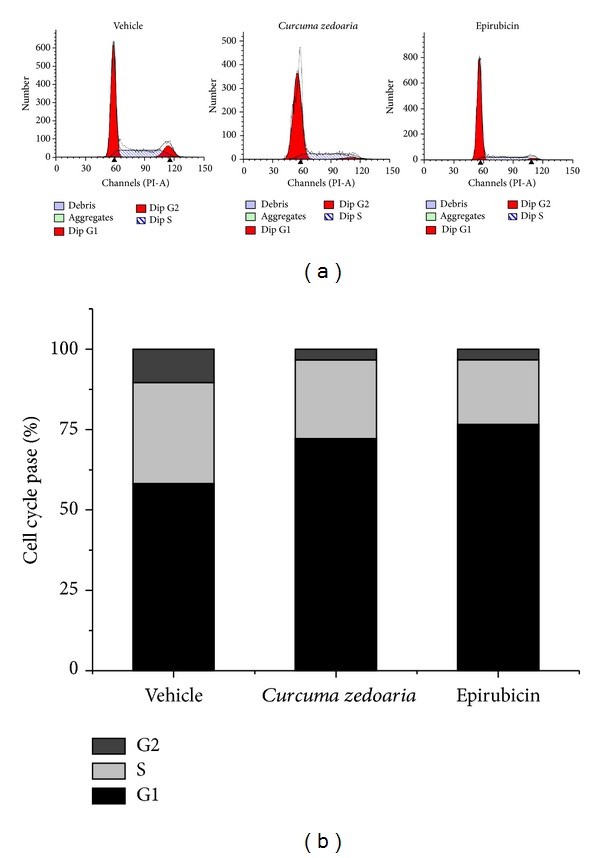
Synchronized MDA-MB-231 cells were treated with petroleum ether extracts of* Curcuma zedoaria* or Epirubicin for 24 hours, and the fraction of cells in each phase of cell cycle was evaluated by flow cytometry.

**Figure 4:**
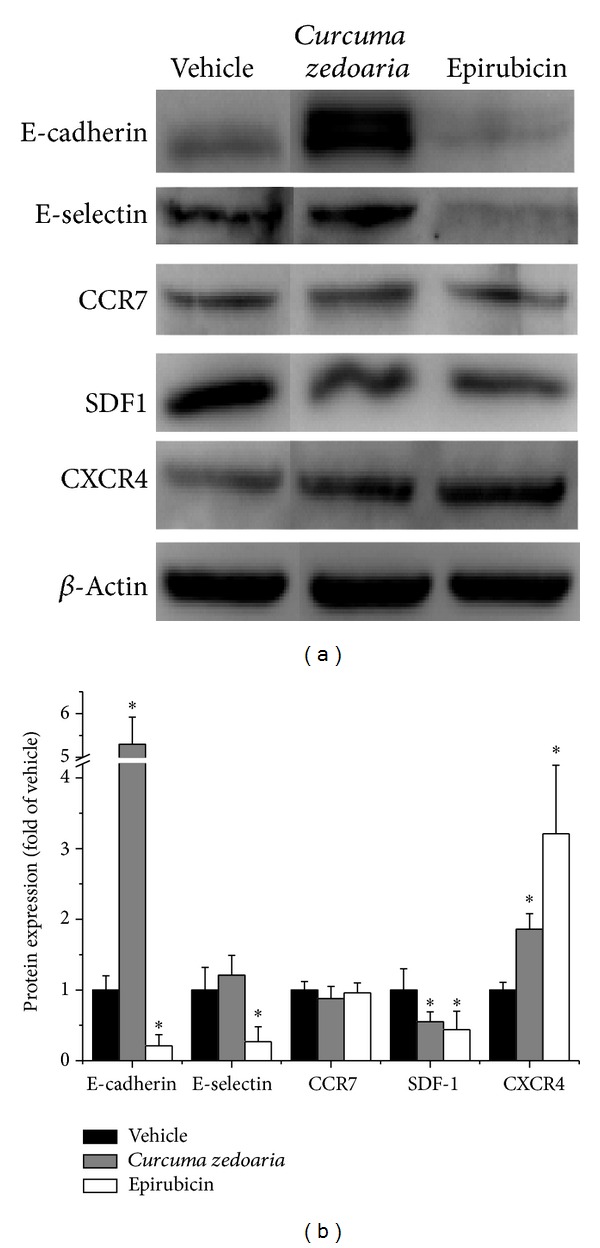
Effect of petroleum ether extracts of* Curcuma zedoaria* (300 *μ*g/mL) and Epirubicin (1 *μ*g/mL) on protein E-cadherin, E-selectin, CCR7, SLC, SDF-1, and CXCR4 expression after 24 hours. Bars represent mean ± SD of three individual experiments. *P < 0.05. Beta-actin was used as a loading control.

**Figure 5:**
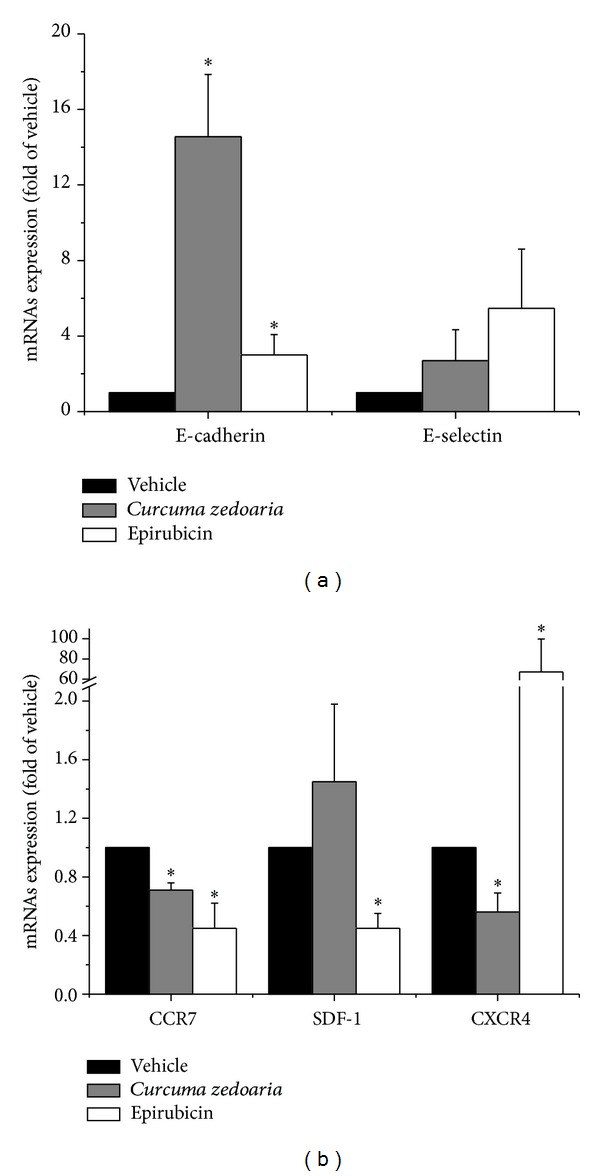
The level of adhesion molecules E-cadherin and E-selectin mRNA expression (a) and chemokines molecules CCR7, SLC, SDF-1, and CXCR4 (b) after 24 hours in different groups by using RT-PCR analysis. *P < 0.01 compared with control.

Many previous studies support the use of* Curcuma zedoaria* rhizomes in traditional medicine for the treatment of cancer-related diseases especially breast, cervical, and colon cancers. As the rhizomes are also widely consumed as salad in food without any known undesirable side effect, it can be assumed that the plant is safe for consumption at the normal dose as food.* Curcuma zedoaria* is therefore a promising dietary agent that holds great promise for use in chemopreventive and chemotherapeutic strategies. We have previously demonstrated that MDA-MB-231 triple-negative breast cancer cells were inhibited by different extracts of* Curcuma zedoaria in vitro* CCK8 assay of cellular viability.* Curcuma zedoaria* plant sample was extracted successively with petroleum ether (PE), ethyl acetate (EA), n-butanol (NB), and water (see Figure 1). The result from assay of cellular viability is that petroleum ether extract of* Curcuma zedoaria* is the strongest in all extracts (Figure 5). In this paper, we analyzed the effect on MDA-MB-231 cells of petroleum ether extracts of* Curcuma zedoaria*.

**Figure 6:**
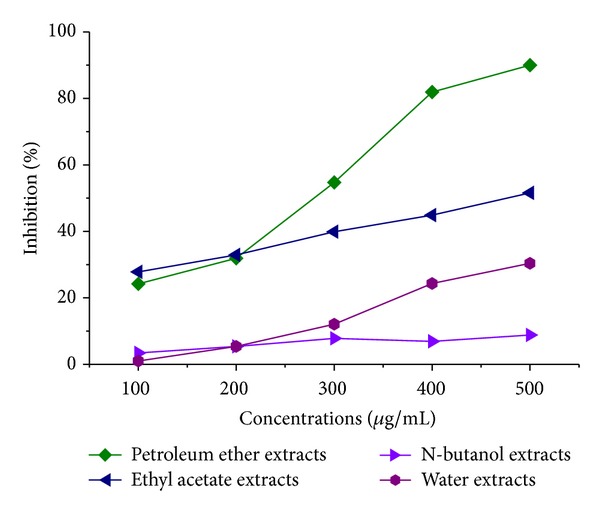
The inhibition rate of the different extracts of* Curcuma zedoaria* was determined by CCK8 assay as described compared with Epirubicin. The values were reported as %.

This study affirms that CCK8 assay has shown that MDA-MB-231 cell viability was significantly affected by exposure to petroleum ether extracts of* Curcuma zedoaria*. The inhibition rate of cell viability was correlated positively with concentration and time. The inhibition rate of petroleum ether extracts of* Curcuma zedoaria* (300 *μ*g/mL concentration) is more than 50%. The analysis of cell cycle progression indicated that petroleum ether extracts of* Curcuma zedoaria* produce an arrest in the G0/G1 phase of cell cycle after 24 h of treatment. Consistent with these observations, petroleum ether extracts of* Curcuma zedoaria* inhibited the active DNA synthesis of MDA-MB-231 cells. From above studies, a similar result was also observed in Epirubicin (see Figure 6).

Why were MDA-MB-231 cell viability and DNA synthesis inhibited by petroleum ether extracts of* Curcuma zedoaria*? Further studies that provide data leading to mechanisms of antitumor are now underway. In breast cancer patients, metastases remain a major cause of disease morbidity and mortality. Breast cancer metastases frequently follow a pattern of dissemination in humans that results in the formation of lesions in the lymph nodes, lungs, liver, and bone marrow [15, 16]. Cross talk between cancer cells and their microenvironment is considered an essential event in tumorigenesis, invasion, and metastasis [17, 18]. Specifically, interactions between transformed epithelial cells and their surrounding stroma may decide the fate of evolving cancers [19], since signals from the microenvironment profoundly influence the survival and migration of cancer cells [20]. We investigated the effects of petroleum ether extracts of* Curcuma zedoaria* on some cell chemokines and adhesion molecules.

Multivariate analysis showed that the combination of E-cadherin-negative and Ki67-positive expression was strongly predictive of poor overall survival in TNBC patients receiving adjuvant chemotherapy [21]. The mechanisms responsible for the chemosensitivity of TNBC with E-cadherin-negative and Ki67-positive expression remain to be determined. Loss of E-cadherin induces epithelial-to-mesenchymal transition (EMT). EMT is a key step toward cancer metastasis. Ahmed et al. reported the close relationship between EMT and the cancer stem cell-like phenotype in response to chemoresistance [22]. Also, other studies have shown that Snail, Slug, and Notch signaling, as EMT markers, were correlated with chemoresistance. These findings suggested that one of the possible mechanisms by which chemosensitivity is reduced in patients with TNBC with loss of E-cadherin expression may involve EMT signaling [23, 24]. In contrast, several studies have reported that E-cadherin-dependent intercellular adhesion enhances chemoresistance [25–27]. Fortunately, the protein and mRNA expression of E-cadherin were significantly increased after treatment with petroleum ether extracts of* Curcuma zedoaria* from our results.

Increasing evidence shows that CXCR4 and its ligand stromal-derived factor-1 (SDF-1*α*, also known as CXCL12) may play a critical role in the organ-selective process of tumorigenesis and metastasis including those observed in breast cancers [28–30]. CXCR4 expression has been established as a prognostic marker in many cancer cell types including breast carcinomas [31–33], and the SDF-1*α*-CXCR4 signaling axis has been associated with breast cancer metastasis [34, 35]. The SDF-1*α*-CXCR4 interaction promotes tumor progression by several possible mechanisms [17, 36, 37]. Several novel CXCR4 antagonists have shown promising* in vitro* anticancer activity in several tumor cell types, including those derived from breast. Furthermore, using animal tumor models, CXCR4 antagonists have* in vivo* anticancer activity as well [38, 39]. The results are in agreement with the reports, showing that the expression of proteins SDF-1, CCR7 mRNA, and CXCR4 mRNA was significantly decreased after treatment with petroleum ether extracts of* Curcuma zedoaria*. But the expression of protein CXCR4 was increased in Western blot assays. The absence of mRNA-protein correlation for a subset of investigated genes suggests that the relation between mRNA and protein is not strictly linear, but it has a more intrinsic and complex dependence. The reason may be that mRNA levels have come down after intervention, expression of constitutive protein is regulated higher by intracellular activation factor, or the expression of protein is lag.

As we know, triple-negative breast cancer (TNBC), which is characterized by negativity for estrogen receptor, progesterone receptor, and human epidermal growth factor receptor 2 (HER2) is a high risk breast cancer that lacks specific targets for treatment selection. Chemotherapy is, therefore, the primary systemic modality used in the treatment of this disease, but reliable parameters to predict the chemosensitivity of TNBC have not been clinically available. Therefore, combination treatment with a nontoxic drug which can improve the TNBC prognosis would be advantageous. Data obtained in our experiments indicate that petroleum ether extracts of* Curcuma zedoaria* are possibly one of antineoplastic parts of the plant. Nevertheless, further investigations are necessary to validate its therapeutic claims and to determine the active ingredient of* Curcuma zedoaria*. It needs to be further investigated both* in vivo* and* in vitro*.

## 5. Conclusion

From above studies, results showed that MDA-MB-231 cells were inhibited by petroleum ether extracts of* Curcuma zedoaria*. This preliminary study and its data persuade us to focus on inhibition TNBC cell of petroleum ether extracts of* Curcuma zedoaria* and investigating further on animal models for* in vivo* evaluation.

## Figures and Tables

**Table 1:** The various concentrations of petroleum ether extracts of *Curcuma zedoaria in vitro* CCK8 assay of cellular viability.

Groups	Concentrations (*μ*g/mL)	*n*	OD value (mean ± SD)	Inhibition rate (%)
Vehicle	—	3	1.49 ± 0.22	0

Epirubicin	0.25	3	0.85 ± 0.12*	49.2
	0.5	3	0.74 ± 0.13**	58.3
	1.0	3	0.67 ± 0.10**	63.8
	2.0	3	0.59 ± 0.02**	69.5
	4.0	3	0.39 ± 0.01**	85.8

*Curcuma zedoaria *	100	3	1.19 ± 0.10*	24.2
	200	3	1.09 ± 0.09**	31.9
	300	3	0.80 ± 0.12**	54.7
	400	3	0.45 ± 0.12**	81.9
	500	3	0.35 ± 0.03**	90.0

Values are average of triplicate experiment and are represented as mean ± SD. *P < 0.05, **P < 0.01 compared with vehicle.

**Table 2:** Effect of petroleum ether extracts of *Curcuma zedoaria  in vitro* cell cycle on progression.

Group	Cell cycle pase (%)		
	G_1_	S	G_2_
Vehicle	58.23 ± 2.02	31.39 ± 1.78	10.37 ± 0.94
Epirubicin	76.60 ± 1.21**	20.11 ± 1.40**	3.30 ± 0.49**
Petroleum ether extracts of *Curcuma zedoaria *	72.21 ± 1.40**	24.43 ± 1.18**	3.35 ± 0.26**

Values are average of triplicate experiment and are represented as mean ± SD.

**P < 0.01 compared with control.

**Table 3:** Effect of petroleum ether extracts of *Curcuma zedoaria* (300 *μ*g/mL) on expression level of E-cadherin, E-selectin, CCR7, SLC, SDF-1, and CXCR4 mRNA after 24 hours.

mRNAs	The relative expression of mRNA (compared to control)		
	Vehicle	Epirubicin	*Curcuma zedoaria *
E-cadherin	1 ± 0	3.00 ± 1.08*	14.55 ± 3.34^∗#^
E-selectin	1 ± 0	5.47 ± 3.13	2.69 ± 1.64
CCR7	1 ± 0	0.45 ± 0.17*	0.71 ± 0.057*
SDF-1	1 ± 0	0.45 ± 0.10*	1.08 ± 0.14
CXCR4	1 ± 0	67.08 ± 32.64*	0.56 ± 0.13^∗#^

Values are average of triplicate experiment and are represented as mean ± SD. *P < 0.01 compared with control, ^∗#^compared with Epirubicin.
